# Supplementary material for: Non-antibiotic medication use in an Indonesian community cohort 0–18 months of age
Source: PLoS One. 2020 Nov 18;15(11):e0242410. doi: 10.1371/journal.pone.0242410 (PMC7673523; doi:10.1371/journal.pone.0242410)
Supplement: S1 Checklist — (DOCX) [file pone.0242410.s001.docx]

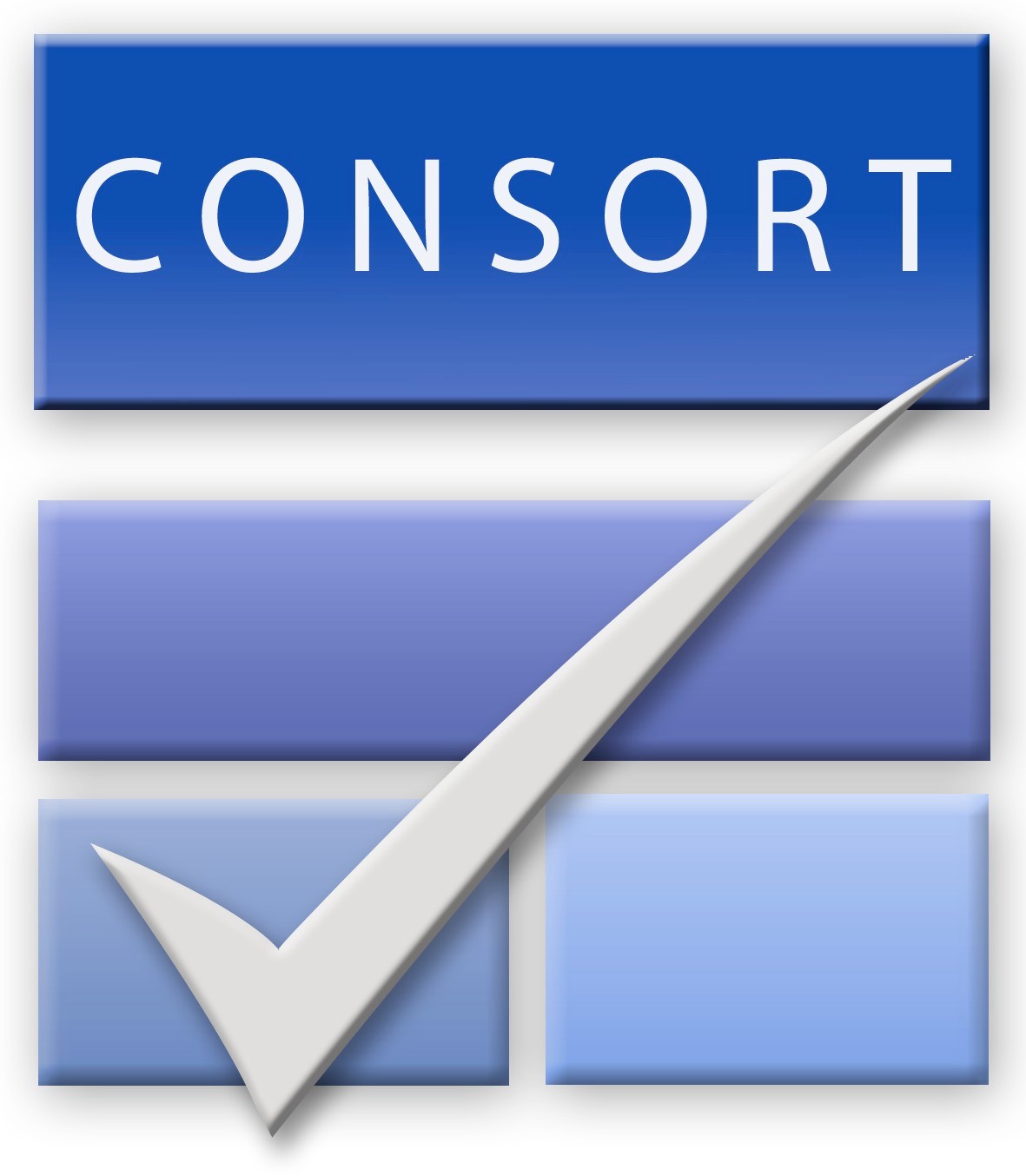
**CONSORT 2010 checklist of information to include when reporting a randomised trial***

| **Section/Topic** | **Item No** | | | **Checklist item** | **Reported on page No** |  |
| --- | --- | --- | --- | --- | --- | --- |
| **Title and abstract** | 1a | | | Identification as a randomised trial in the title | Identified in the original trial article |  |
|  | 1b | | | Structured summary of trial design, methods, results, and conclusions (for specific guidance see CONSORT for abstracts) | p.2, lines 48-71 |  |
| **Introduction**  Background and | 2a | | | Scientific background and explanation of rationale | Described in the original trial article |  |
| objectives | 2b | | | Specific objectives or hypotheses | p.3, lines 91-94 |  |
| **Methods**  Trial design | 3a | | | Description of trial design (such as parallel, factorial) including allocation ratio | p.3-4, lines 97-136 |  |
|  | 3b | | | Important changes to methods after trial commencement (such as eligibility criteria), with reasons | NA |  |
| Participants | 4a | | | Eligibility criteria for participants | p.3, lines 116-118 |  |
|  | 4b | | | Settings and locations where the data were collected | p.3, lines 106-108 |  |
| Interventions | 5 | | | The interventions for each group with sufficient details to allow replication, including how and when they were | p.4, lines 122-131 |  |
|  |  | | | actually administered |  |  |
| Outcomes | 6a | | | Completely defined pre-specified primary and secondary outcome measures, including how and when they | Described in the original trial article |  |
|  |  | | | were assessed |  |  |
|  | 6b | | | Any changes to trial outcomes after the trial commenced, with reasons | NA |  |
| Sample size | 7a | | | How sample size was determined | p.4, lines 138-145 |  |
|  | 7b | | | When applicable, explanation of any interim analyses and stopping guidelines | NA |  |
| Randomisation: |  | | |  |  |  |
| Sequence | 8a | | | Method used to generate the random allocation sequence | p.4, lines 132-133 |  |
| generation | 8b | | | Type of randomisation; details of any restriction (such as blocking and block size) | p.4, lines 132-133 |  |
| Allocation | 9 | | | Mechanism used to implement the random allocation sequence (such as sequentially numbered containers), | Described in the original trial article |  |
| concealment |  | | | describing any steps taken to conceal the sequence until interventions were assigned |  |  |
| mechanism |  | | |  |  |  |
| Implementation | 10 | | | Who generated the random allocation sequence, who enrolled participants, and who assigned participants to | Described in the original trial article |  |
|  |  | | | interventions |  |  |
| Blinding | 11a | | | If done, who was blinded after assignment to interventions (for example, participants, care providers, those assessing outcomes) and how | p.4, lines 133-136 |  |
|  | | | 11b | If relevant, description of the similarity of interventions | | NA |
| Statistical methods | | | 12a | Statistical methods used to compare groups for primary and secondary outcomes | | p.5, lines 184-188 |
|  | | | 12b | Methods for additional analyses, such as subgroup analyses and adjusted analyses | | p.5, lines 184-188 |
| **Results**  Participant flow (a | | | 13a | For each group, the numbers of participants who were randomly assigned, received intended treatment, and | |  |
| diagram is strongly | | |  | were analysed for the primary outcome | | Fig. 1 |
| recommended) | | | 13b | For each group, losses and exclusions after randomisation, together with reasons | | Fig. 1 |
| Recruitment | | | 14a | Dates defining the periods of recruitment and follow-up | | p.3, line 106 |
|  | | | 14b | Why the trial ended or was stopped | | NA |
| Baseline data | | | 15 | A table showing baseline demographic and clinical characteristics for each group | | p.5 (Table-1) |
| Numbers analysed | | | 16 | For each group, number of participants (denominator) included in each analysis and whether the analysis was | |  |
|  | | |  | by original assigned groups | | p.5 (Table-1) |
| Outcomes and | | | 17a | For each primary and secondary outcome, results for each group, and the estimated effect size and its | |  |
| estimation | | |  | precision (such as 95% confidence interval) | | p.5 (Table-1) |
|  | | | 17b | For binary outcomes, presentation of both absolute and relative effect sizes is recommended | | p.5, Table-1 |
| Ancillary analyses | | | 18 | Results of any other analyses performed, including subgroup analyses and adjusted analyses, distinguishing | |  |
|  | | |  | pre-specified from exploratory | | NA |
| Harms | | | 19 | All important harms or unintended effects in each group (for specific guidance see CONSORT for harms) | | NA |
| **Discussion**  Limitations | | | 20 | Trial limitations, addressing sources of potential bias, imprecision, and, if relevant, multiplicity of analyses | | NA |
| Generalisability | | | 21 | Generalisability (external validity, applicability) of the trial findings | | p.13, lines 327-329 |
| Interpretation | | | 22 | Interpretation consistent with results, balancing benefits and harms, and considering other relevant evidence | | p.11-15 (Discussions) |
| **Other information**  Registration | | | 23 | Registration number and name of trial registry | | p.3, line 100 |
| Protocol | | | 24 | Where the full trial protocol can be accessed, if available | | p.3, line 100 |
| Funding | | | 25 | Sources of funding and other support (such as supply of drugs), role of funders | | Described separately |

*We strongly recommend reading this statement in conjunction with the CONSORT 2010 Explanation and Elaboration for important clarifications on all the items. If relevant, we also recommend reading CONSORT extensions for cluster randomised trials, non-inferiority and equivalence trials, non-pharmacological treatments, herbal interventions, and pragmatic trials. Additional extensions are forthcoming: for those and for up to date references relevant to this checklist, see [www.consort-statement.org.](http://www.consort-statement.org/)
