# Supplementary material for: Non-antibiotic medication use in an Indonesian community cohort 0–18 months of age
Source: PLoS One. 2020 Nov 18;15(11):e0242410. doi: 10.1371/journal.pone.0242410 (PMC7673523; doi:10.1371/journal.pone.0242410)
Supplement: S1 Table — (DOCX) [file pone.0242410.s002.docx]

**S1 Table. Therapeutic classes used by trial participants**

| **No.** | **ATC Code** | **Medication classes** | **No. of drug use (%)** | **No. of participants** | **Prevalence rate (per 100 participants)** |
| --- | --- | --- | --- | --- | --- |
|  | N02 | Analgesics/antipyretics | 2280 (30.06) | 1093 | 67.43 |
|  | R06 | Antihistamines for systemic use | 1316 (17.35) | 772 | 47.62 |
|  | R05 | Cough and cold preparations | 1020 (13.45) | 614 | 37.88 |
|  | A11 | Vitamins | 652 (8.59) | 411 | 25.35 |
|  | A07 | Antidiarrheals, intestinal antiinflammary agents | 502 (6.62) | 312 | 19.25 |
|  | A12 | Mineral supplements | 490 (6.46) | 325 | 20.05 |
|  | R03 | Bronchodilators | 287 (3.78) | 185 | 11.41 |
|  | D07 | Corticosteroids, dermatological preparations | 189 (2.49) | 156 | 9.62 |
|  | R01 | Nasal preparations | 178 (2.35) | 150 | 9.25 |
|  | D02 | Emollients and protectives | 145 (1.91) | 123 | 7.59 |
|  | A02 | Drugs for acid related disorders | 80 (1.05) | 69 | 4.26 |
|  | A06 | Drug for constipation | 52 (0.69) | 40 | 2.47 |
|  | H02 | Corticosteroids for systemic use | 46 (0.61) | 38 | 2.34 |
|  | D08 | Antiseptics and disinfectants | 41 (0.54) | 41 | 2.53 |
|  | D01 | Antifungals for dermatological use | 38 (0.50) | 36 | 2.22 |
|  | A03 | Drugs for functional gastrointestinal disorders | 32 (0.42) | 27 | 1.67 |
|  | D11 | Other dermatological preparations | 29 (0.38) | 28 | 1.73 |
|  | N05 | Psycholeptics | 29 (0.38) | 24 | 1.48 |
|  | B05 | Blood substitutes and perfusion solutions | 21 (0.28) | 19 | 1.17 |
|  | B03 | Antianemic preparations | 18 (0.24) | 14 | 0.86 |
|  | N03 | Antiepileptics | 16 (0.21) | 9 | 0.56 |
|  | D04 | Antipruritics, including antihistamines, anesthetics, etc. | 16 (0.21) | 15 | 0.93 |
|  | V03 | All other therapeutic products | 15 (0.20) | 14 | 0.86 |
|  | D06 | Antibiotics and chemotherapeutics for dermatological use | 14 (0.18) | 13 | 0.80 |
|  | D09 | Medicated dressings | 13 (0.17) | 11 | 0.68 |
|  | C01 | Cardiac therapy | 11 (0.15) | 2 | 0.12 |
|  | A04 | Antiemetics and antinauseants | 7 (0.09) | 7 | 0.43 |
|  | A08 | Antiobesity preparations, excluding diet products | 6 (0.08) | 5 | 0.31 |
|  | A13 | Tonics | 6 (0.08) | 5 | 0.31 |
|  | C09 | Agent acting on the renin-angiotensin system | 4 (0.05) | 2 | 0.12 |
|  | C03 | Diuretics | 4 (0.05) | 2 | 0.12 |
|  | C10 | Lipid modifying agents | 4 (0.05) | 4 | 0.25 |
|  | M01 | Antiinflammatory and antirheumatic products | 3 (0.04) | 3 | 0.19 |
|  | S01 | Ophthalmologicals | 3 (0.04) | 3 | 0.19 |
|  | N01 | Anesthetics | 2 (0.03) | 1 | 0.06 |
|  | J02 | Antimycotics for systemic use | 2 (0.03) | 2 | 0.12 |
|  | B01 | Antithrombotic agents | 2 (0.03) | 2 | 0.12 |
|  | A05 | Bile and liver therapy | 2 (0.03) | 1 | 0.06 |
|  | A10 | Drugs used in diabetes | 2 (0.03) | 2 | 0.12 |
|  | P03 | Ectoparasiticides, incl. scabicides, insecticides and repellents | 1 (0.01) | 1 | 0.06 |
|  | V06 | General nutrients | 1 (0.01) | 1 | 0.06 |
|  | J06 | Immune sera and immunoglobulins | 1 (0.01) | 1 | 0.06 |
|  | M03 | Muscle relaxants | 1 (0.01) | 1 | 0.06 |
|  | A16 | Other alimentary tract and metabolism products | 1 (0.01) | 1 | 0.06 |
|  | D03 | Preparations for treatment of wounds and ulcers | 1 (0.01) | 1 | 0.06 |
|  | N06 | Psychoanaleptics | 1 (0.01) | 1 | 0.06 |
|  | G04 | Urologicals | 1 (0.01) | 1 | 0.06 |
|  | C05 | Vasoprotectives | 1 (0.01) | 1 | 0.06 |
