# Supplementary material for: Non-antibiotic medication use in an Indonesian community cohort 0–18 months of age
Source: PLoS One. 2020 Nov 18;15(11):e0242410. doi: 10.1371/journal.pone.0242410 (PMC7673523; doi:10.1371/journal.pone.0242410)
Supplement: S2 Table — (DOCX) [file pone.0242410.s003.docx]

**S2 Table. Type of drugs used by trial participants**

| **No.** | **ATC Code** | **Medication classes** | **No. of drug use (%)** | **No. of participants** | **Prevalence rate (per 100 participants)** |
| --- | --- | --- | --- | --- | --- |
|  | N02BE01 | Paracetamol | 2268 (29.90) | 1093 | 67.43 |
|  | R06AB04 | Chlorpheniramine | 1275 (16.81) | 753 | 46.45 |
|  | R05CA03 | Guaifenesin | 677 (8.92) | 465 | 28.69 |
|  | A12CB01 | Zinc | 414 (5.46) | 294 | 18.14 |
|  | R05CB06 | Ambroxol | 311 (4.10) | 226 | 13.94 |
|  | R03CC02 | Salbutamol | 273 (3.60) | 181 | 11.17 |
|  | A07CA | Oral rehydration salt formulations | 255 (3.36) | 193 | 11.91 |
|  | A11GA01 | Ascorbic acid (vit C) | 227 (2.99) | 179 | 11.04 |
|  | A11HA02 | Vitamin B6 | 185 (2.44) | 140 | 8.64 |
|  | A11EA | Vitamin B complex (unspecified) | 181 (2.39) | 139 | 8.57 |
|  | A07FA51 | Probiotics | 130 (1.71) | 110 | 6.79 |
|  | D07AA02 | Hydrocortisone | 127 (1.67) | 112 | 6.91 |
|  | H02AB02 | Dexamethasone | 117 (1.54) | 97 | 5.98 |
|  | A12AA05 | Calcium | 79 (1.04) | 55 | 3.39 |
|  | D02AX | Other emollients and protectives | 73 (0.96) | 64 | 3.95 |
|  | A02AD01 | Ordinary salt combinations | 70 (0.92) | 62 | 3.82 |
|  | D02AF | Salicyclic acid preparations | 66 (0.87) | 60 | 3.70 |
|  | A07BC30 | Combinations on anti-diarrheals (unspecified) | 60 (0.79) | 57 | 3.52 |
|  | A06AG11 | Laurilsulfate | 42 (0.55) | 34 | 2.10 |
|  | R06AE07 | Cetirizine | 36 (0.47) | 30 | 1.85 |
|  | D07AC01 | Betamethasone | 34 (0.45) | 31 | 1.91 |
|  | R01AD52 | Prednisolone | 34 (0.45) | 28 | 1.73 |
|  | R01BA02 | Pseudoephedrine | 31 (0.41) | 29 | 1.79 |
|  | A03FA03 | Domperidone | 30 (0.40) | 26 | 1.60 |
|  | D01AC02 | Miconazole | 30 (0.40) | 29 | 1.79 |
|  | D11AX | Other dermatological preparations (unspecified) | 29 (0.38) | 28 | 1.73 |
|  | N05BA01 | Diazepam | 22 (0.29) | 18 | 1.11 |
|  | R05DA09 | Dextromethorphan | 20 (0.26) | 18 | 1.11 |
|  | A07AA02 | Nystatin | 20 (0.26) | 20 | 1.23 |
|  | A07BC30 | Kaolin pectin | 18 (0.24) | 18 | 1.11 |
|  | A11BA | Multivitamins, plain | 18 (0.24) | 15 | 0.93 |
|  | H02AB07 | Prednisone | 18 (0.24) | 15 | 0.93 |
|  | A11AA03 | Multivitamins and other minerals (unspecified) | 16 (0.21) | 16 | 0.99 |
|  | D04AX | Other antipruritics | 16 (0.21) | 15 | 0.93 |
|  | N03AA02 | Phenobarbital | 16 (0.21) | 10 | 0.62 |
|  | V03AN01 | Oxygen | 15 (0.20) | 14 | 0.86 |
|  | A11DA01 | Vitamin B1 | 15 (0.20) | 14 | 0.86 |
|  | D08A | Antiseptics and disinfectants | 14 (0.18) | 14 | 0.86 |
|  | D07AC13 | Mometasone | 14 (0.18) | 12 | 0.74 |
|  | D06BB03 | Aciclovir | 13 (0.17) | 12 | 0.74 |
|  | A07BC04 | Attapulgite | 13 (0.17) | 12 | 0.74 |
|  | D09AA09 | Povidone-iodine | 13 (0.17) | 11 | 0.68 |
|  | B05CB01 | Sodium chloride | 13 (0.17) | 12 | 0.78 |
|  | D07AB08 | Desonide | 12 (0.16) | 12 | 0.74 |
|  | R01BA51 | Phenylpropanolamine | 11 (0.15) | 10 | 0.62 |
|  | D08AX06 | Potassium permanganate | 11 (0.15) | 11 | 0.68 |
|  | R05X | Combinations of more than one cough preparation (unspecified) | 10 (0.13) | 10 | 0.62 |
|  | B03BB01 | Folic acid | 9 (0.12) | 7 | 0.43 |
|  | R03CA02 | Ephedrine | 8 (0.11) | 7 | 0.43 |
|  | N02BB02 | Metamizole sodium | 8 (0.11) | 8 | 0.49 |
|  | A11CA | Vitamin A, plain | 8 (0.11) | 8 | 0.49 |
|  | H02AB04 | Methylprednisolone | 7 (0.09) | 7 | 0.43 |
|  | A04AA01 | Ondansetron | 7 (0.09) | 7 | 0.43 |
|  | D08AX | Other antiseptics and disinfectants | 7 (0.09) | 7 | 0.43 |
|  | R01BA53 | Phenylephrine | 6 (0.08) | 5 | 0.31 |
|  | A13A | Tonics | 6 (0.08) | 5 | 0.31 |
|  | A06AB02 | Bisacodyl | 5 (0.07) | 4 | 0.25 |
|  | C01CA07 | Dobutamine | 5 (0.07) | 2 | 0.12 |
|  | A02BA02 | Ranitidine | 5 (0.07) | 5 | 0.31 |
|  | B05CB04 | Sodium bicarbonate | 5 (0.07) | 4 | 0.25 |
|  | R03DA05 | Aminophylline | 4 (0.05) | 4 | 0.25 |
|  | C09AA01 | Captopril | 4 (0.05) | 2 | 0.12 |
|  | C10AC01 | Colestyramine | 4 (0.05) | 4 | 0.25 |
|  | C01CA04 | Dopamine | 4 (0.05) | 2 | 0.12 |
|  | C03CA01 | Furosemide | 4 (0.05) | 2 | 0.12 |
|  | M01AE01 | Ibuprofen | 4 (0.05) | 4 | 0.25 |
|  | B03AD | Iron preparations | 4 (0.05) | 4 | 0.25 |
|  | D01AC08 | Ketoconazole | 4 (0.05) | 4 | 0.25 |
|  | A07DA03 | Loperamide | 4 (0.05) | 3 | 0.19 |
|  | B03BA05 | Mecobalamin | 4 (0.05) | 2 | 0.12 |
|  | A07BC | Other intestinal adsorbents | 4 (0.05) | 2 | 0.12 |
|  | R03DA04 | Theophylline | 4 (0.05) | 3 | 0.19 |
|  | M01AB16 | Aceclofenac | 3 (0.04) | 3 | 0.19 |
|  | A02BA01 | Cimetidine | 3 (0.04) | 3 | 0.19 |
|  | R06AA02 | Diphenhydramine | 3 (0.04) | 3 | 0.19 |
|  | D08AA01 | Ethacridine lactate | 3 (0.04) | 3 | 0.19 |
|  | N05CD08 | Midazolam | 3 (0.04) | 3 | 0.19 |
|  | D01A | Antifungals for topical use | 2 (0.03) | 2 | 0.12 |
|  | R03BA02 | Budesonide | 2 (0.03) | 2 | 0.12 |
|  | D08AE05 | Chloroxylenol | 2 (0.03) | 2 | 0.12 |
|  | C01CA24 | Epinephrine | 2 (0.03) | 1 | 0.06 |
|  | R03BA05 | Fluticasone | 2 (0.03) | 2 | 0.12 |
|  | B01AB01 | Heparin | 2 (0.03) | 2 | 0.12 |
|  | A03FA01 | Metoclopramide | 2 (0.03) | 1 | 0.06 |
|  | D08AE03 | Phenol | 2 (0.03) | 2 | 0.12 |
|  | D08AE02 | Policresulen | 2 (0.03) | 2 | 0.12 |
|  | D02AA | Silicone products | 2 (0.03) | 2 | 0.12 |
|  | A06AB08 | Sodium picosulfate | 2 (0.03) | 2 | 0.12 |
|  | A02BX02 | Sucralfate | 2 (0.03) | 2 | 0.12 |
|  | S01ED01 | Timolol | 2 (0.03) | 2 | 0.12 |
|  | R01AD11 | Triamcinolone | 2 (0.03) | 2 | 0.12 |
|  | A05AA02 | Ursodeoxycholic acid | 2 (0.03) | 1 | 0.06 |
|  | N03AG01 | Valproic acid | 2 (0.03) | 1 | 0.06 |
|  | A11JB | Vitamins with minerals | 2 (0.03) | 1 | 0.06 |
|  | S01EC01 | Acetazolamide | 1 (0.01) | 1 | 0.06 |
|  | B05BA01 | Amino acid | 1 (0.01) | 1 | 0.06 |
|  | R05CB02 | Bromhexine | 1 (0.01) | 1 | 0.06 |
|  | N05AA01 | Chlorpromazine | 1 (0.01) | 1 | 0.06 |
|  | R05DA04 | Codeine | 1 (0.01) | 1 | 0.06 |
|  | D03AX03 | Dexpanthenol | 1 (0.01) | 1 | 0.06 |
|  | A06A | Drugs for constipation | 1 (0.01) | 1 | 0.06 |
|  | D02A | Emollients and protectives | 1 (0.01) | 1 | 0.06 |
|  | B05AX01 | Erythrocytes | 1 (0.01) | 1 | 0.06 |
|  | B05BA02 | Fat emulsions | 1 (0.01) | 1 | 0.06 |
|  | N01AH01 | Fentanyl | 1 (0.01) | 1 | 0.06 |
|  | D07XC05 | Fluocortolone | 1 (0.01) | 1 | 0.06 |
|  | A06AG04 | Glycerol | 1 (0.01) | 1 | 0.06 |
|  | D01BA01 | Griseofulvin | 1 (0.01) | 1 | 0.06 |
|  | J06BA02 | Immunoglobulins, normal human, for intravascular administration | 1 (0.01) | 1 | 0.06 |
|  | V06CA | Infant formulas | 1 (0.01) | 1 | 0.06 |
|  | A10A | Insulins and analogues | 1 (0.01) | 1 | 0.06 |
|  | A16AA01 | Levocarnitine | 1 (0.01) | 1 | 0.06 |
|  | A06AA51 | Liquid paraffin | 1 (0.01) | 1 | 0.06 |
|  | R06AX15 | Mebhydrolin | 1 (0.01) | 1 | 0.06 |
|  | A10BA02 | Metformin | 1 (0.01) | 1 | 0.06 |
|  | M03BC51 | Orphenadrine | 1 (0.01) | 1 | 0.06 |
|  | P03AC04 | Permethrin | 1 (0.01) | 1 | 0.06 |
|  | N03AB02 | Phenytoin | 1 (0.01) | 1 | 0.06 |
|  | N06BX03 | Piracetam | 1 (0.01) | 1 | 0.06 |
|  | R06AD02 | Promethazine | 1 (0.01) | 1 | 0.06 |
|  | N01AB08 | Sevoflurane | 1 (0.01) | 1 | 0.06 |
|  | G04BE03 | Sildenafil | 1 (0.01) | 1 | 0.06 |
|  | D06BA01 | Silver sulfadiazine | 1 (0.01) | 1 | 0.06 |
|  | D01AE15 | Terbinafine | 1 (0.01) | 1 | 0.06 |
|  | B03BA05 | Vitamin B12 | 1 (0.01) | 1 | 0.06 |
