# Supplementary material for: Non-antibiotic medication use in an Indonesian community cohort 0–18 months of age
Source: PLoS One. 2020 Nov 18;15(11):e0242410. doi: 10.1371/journal.pone.0242410 (PMC7673523; doi:10.1371/journal.pone.0242410)
Supplement: Attachment — Submitted filename: Response to reviewers (v1.1_31Dec2019).docx [file pone.0242410.s005.docx]

December 31, 2019

Subject: Submission of revised paper *Non-antibiotic Medication Use in an Indonesian Community Cohort 0-18 Months of Age*

[Manuscript number: PONE-D-19-26757]

Dear Academic Editor and Reviewers

Thank you for spending your valuable time to read our previous manuscript and giving insightful suggestions to help us improve the quality of our manuscript.

Along with this letter are the Academic Editor and Reviewers comments, followed by our point-by-point response in addition to making the changes in the revised manuscript. Responses to the Academic Editor and Reviewers’ comments are typed in blue font, and the changes made in the manuscript are marked using track changes. Each author has given approval to the final form of this revision.

In this cover letter, we also would like to propose the following updated competing interests statement as suggested by Academic Editor:

“The authors have the following interests: NSB is a paid employee of PT Biofarma. PT Biofarma is a state-owned company (owned by Indonesia government) that provides all vaccines for Indonesia National Immunization Program. Additionally, PT Biofarma was one of **the non-commercial** funders for the RV3-BB Phase IIB trial. **No consultation was done to the funder relating to the trial.** The trial was a phase IIB trial and while the RV3-BB rotavirus vaccine is not yet marketed, this vaccine is being developed with the intention for eventual usage in the national immunization program. This does not alter our adherence to PLOS ONE policies on sharing data and materials.”

We believe that the revision on our manuscript will improve the quality of our paper. We look forward to hearing from you in due course.

Sincerely,

Jarir At Thobari, MD, DPharm, PhD

Department of Pharmacology and Therapy and Pediatric Research Office (PRO) Faculty of Medicine, Public Health, and Nursing

Universitas Gadjah Mada

On behalf of all authors.

**Journal Requirements:**

1. When submitting your revision, we need you to address these additional requirements.

Please ensure that your manuscript meets PLOS ONE's style requirements, including those for file naming. The PLOS ONE style templates can be found at

[http://www.journals.plos.org/plosone/s/file?id=wjVg/PLOSOne_formatting_sample_main_body.pdf](http://www.journals.plos.org/plosone/s/file?id=wjVg/PLOSOne_formatting_sample_main_body.pdf) and [http://www.journals.plos.org/plosone/s/file?id=ba62/PLOSOne_formatting_sample_title_authors_affiliations.pdf](http://www.journals.plos.org/plosone/s/file?id=ba62/PLOSOne_formatting_sample_title_authors_affiliations.pdf)

**Response**: Thank you for enclosing the PLOS ONE style templates which make it easier for us to comply with the requirement. We have read and followed the PLOS ONE’s style requirements carefully.

2. Thank you for stating the following in the Competing Interests section:

I have read the journal's policy and the authors of this manuscript have the following competing interests: NSB is a paid employee of PT Biofarma. PT Biofarma is a state-owned company (owned by Indonesia government) that provides all vaccines for Indonesia National Immunization Program. Additionally, PT Biofarma was one of the funders for the RV3-BB Phase IIB trial. The trial was a phase IIB trial and while the RV3-BB rotavirus vaccine is not yet marketed, this vaccine is being developed with the intention for eventual usage in the national immunization program. This does not alter our adherence to PLOS ONE policies on sharing data and materials.

We note that you received funding from a commercial source: PT Biofarma.

Please provide an amended Competing Interests Statement that **explicitly states this commercial funder, along with any other [relevant declarations](http://www.plosone.org/static/competing.action) relating to employment, consultancy, patents, products in development, marketed products, etc**.

**Response:** We apologize for not making this sufficiently clear in the first submission. As a state-owned company that provides all vaccines for Indonesia National Immunization Program, PT Biofarma was one of the non-commercial funders in the development of RV3-BB rotavirus vaccine. The vaccine is developed for eventual usage in the national immunization program. Employment, patents, products in development and marketed products have been described in the previous Competing Interests Statement. Moreover, no consultation was done to the funder relating to the trial.

In addition to this clarification, we would like to propose the following updated competing interests statement:

“The authors have the following interests: NSB is a paid employee of PT Biofarma. PT Biofarma is a state-owned company (owned by Indonesia government) that provides all vaccines for Indonesia National Immunization Program. Additionally, PT Biofarma was one of **the non-commercial** funders for the RV3-BB Phase IIB trial. **No consultation was done to the funder relating to the trial.** The trial was a phase IIB trial and while the RV3-BB rotavirus vaccine is not yet marketed, this vaccine is being developed with the intention for eventual usage in the national immunization program. This does not alter our adherence to PLOS ONE policies on sharing data and materials.”

Review Comments to the Author

Please use the space provided to explain your answers to the questions above. You may also include additional comments for the author, including concerns about dual publication, research ethics, or publication ethics. (Please upload your review as an attachment if it exceeds 20,000 characters)

Reviewer #1:

1. Table 5: column 5 does not show % of participants

**Response:** Thank you for noting this. We have deleted the “%” from Table 5 (now namely table 6) since the prevalence rate was per 100 participants (similar with %).

2. Table 6: whats the difference between other infections disease group and other viral infections.Are viral infections included in other infections.I think it can be combined to one group as other infections (gabung aja)

**Response:** Thank you for your suggestion. We have combined the “other viral infections” and “other infections diseases”. We previously separated these groups of disease because we wanted to look for antimicrobial use pattern among different infectious diseases (in the previous article).

Therefore, the following parts of the manuscript have been revised

a) Line 157: we changed the “13 disorders” into “12 disorders”

b) In Table 7, we combined the “other viral infections” and “other infections diseases” into “other infections”

Reviewer #2: There are two points to be modified in the coming revision:

1. Reading through the manuscript, I found only tables presented in a comprehensive way but no any figures demonstrated. I would recommend the authors present their results in Figures (e.g., pie charts).

**Response:** As suggested by the reviewer, we have substituted the previously named Table 6 (in initial manuscript) with two figures.

a) Fig 2. represents the reason for non-antibiotic medication use in bar chart.

b) Figure 3 represents the five main therapeutic classes used based on the reason for non-antibiotic medication use

These two new figures are submitted in separated .tif files as requested by Plos One submission guideline.

2. Regarding the statistical assessments on the data collected, it is clear that there are only very descriptive metrics calculated while the more comparative statistics should be performed to get insights of the cohorts. It is also necessary to include more sophisticated statistics for a report on cohort studies.

**Response:** Thank you for noting this. As suggested by the reviewer, we have calculated a stepwise linear regression to predict the number of drug regimen used based on: the reason for medication use, drug formulation, outpatient/inpatient setting, and participant’s age when using the medication (month). Gender and intervention group were excluded after the regression since the p-value >0.05. We described this additional statistical analysis in:

a) Line 189-191 (statistical analysis – method section) : “A stepwise linear regression was performed to predict the number of non-antibiotic drug regimen based on: the reason of medication use, drug formulation, outpatient/inpatient setting, and the age of participant when using the medication.:

b) Table 4. Predictors for the number of drug regimen after regression

c) Line 231-245 (Factors associated with the number of non-antibiotic drug regimen - Results Section)

A stepwise linear regression was calculated to predict the number of drug regimen used based on: the reason for medication use, drug formulation, outpatient/inpatient setting, and participant’s age when using the medication (month). Gender and intervention group were excluded (*p*>0.05) following the regression. A significant regression equation was found (p = 0.00) with an R^2^ of 0.25. Participant’s predicted number of drug regimen is equal to 2.71 – 0.13 (reason for medication use) - 0.46 (route of administration) + 0.17 (outpatient/inpatient setting), with reasons for medication use is coded as 1= Respiratory; 2= Gastrointestinal; 3= Ocular; 4= Skin; 5= Ear; 6= Genitourinary; 7= Nervous system; 8= Metabolism and nutrition; 9= Unspecified pyrexia; 10= Other infections; 11= Other diseases. Route of administration is coded as 1= Oral; 2= Topical; 3= Other formulation. Outpatient is coded 0; and inpatient is 1.

These results indicate that respiratory illnesses, oral drug formulation, and inpatient setting increase the number of drug regimen. Participant’s age when using the medication is measured in month. The number of drug regimen increased 0.01 for each 1 month of participant’s age.

d) Discussion section

Lines 335-337: “This may explains why oral formulation is associated with higher number of drug regimen in this study (Table 4).”

Lines 352-353: “This also may explains why the participant’s age when using medication in this study is associated with the number of drug regimen (Table 4).”

Lines 358-363: “Respiratory illnesses, oral drug formulation and inpatient setting were the main factors to increase the number of non-antibiotic drug regimen. Respiratory illness tends to develop many symptoms including fever, rhinorrhea, cough, sneezing, and sore throat. This multi-symptom condition may encourage child caretakers to self-medicate the children with multiple over-the-counter (OTC) cough and cold medications (CCMs). Hospitalized child may also indicate a more complex diagnosis to be treated, thus more medications may be required [10]”
