# Supplementary material for: Non-antibiotic medication use in an Indonesian community cohort 0–18 months of age
Source: PLoS One. 2020 Nov 18;15(11):e0242410. doi: 10.1371/journal.pone.0242410 (PMC7673523; doi:10.1371/journal.pone.0242410)
Supplement: Attachment — Submitted filename: Response to reviewers_2nd revision (ver2.1_15Jun2020).docx [file pone.0242410.s006.docx]

June 15, 2020

Subject: Submission of revised paper *Non-antibiotic Medication Use in an Indonesian Community Cohort 0-18 Months of Age*

[Manuscript number: PONE-D-19-26757]

Dear Academic Editor and Reviewers

Thank you for spending your valuable time to read our previously revised manuscript and keep giving insightful suggestions to help us improve the quality of our manuscript.

Along with this letter are the Reviewers comments, followed by our point-by-point response in addition to making the changes in the revised manuscript. Responses to the Reviewers’ comments are typed in blue font, and the changes made in the manuscript are marked using track changes. Each author has given approval to the final form of this revision.

We believe that the revision on our manuscript will improve the quality of our paper. We look forward to hearing from you in due course.

Sincerely,

Jarir At Thobari, MD, DPharm, PhD

Department of Pharmacology and Therapy and Pediatric Research Office (PRO)

Faculty of Medicine, Public Health, and Nursing

Universitas Gadjah Mada

On behalf of all authors

2. Reviewer #1: one additional comment-How statistics were done for table 4.Please elaborate as its not easy to understand

**Response:** We thank the reviewer for noting this issue. After re-considering the best model to explore the factors that influenced the number of drug regimen in our study, we decided that instead of using a linear regression as in our previous manuscript, a logistic regression is better to provide the RR. Therefore, at the following lines we have added some changes:

Line 230-238: “A logistic regression was performed to explore the relationship between the reason of medication use, drug formulation, outpatient/inpatient setting, and the age of participant when using the medication with the number of non-antibiotic drug regimen.“

We also have revised the Table 4 and lines 348-355 (discussion section).

3. Reviewer #3: Abstract: Early on in the abstract make the age range of participants clear.

**Response**: Thank you for noting this, now we have added “aged 0-18 months” at line 53

“A post-hoc study of the RV3-BB Phase IIb trial **to children aged 0-18 months** which was conducted in Indonesia during January 2013 to July 2016. Any concomitant medication use and health events among 1621 trial participants during the 18 months of follow-up were documented. Information on medication use included the frequency, formulation, indication, duration of usage, number of regimens, medication types, and therapeutic classes.”

4. What was the original aim of the study before the concomitant meds were looked at in this paper?

**Response**: The original aim of the study was primarily to evaluate the efficacy of RV3-BB vaccine against severe rotavirus gastroenteritis in children aged up to 18 months. This explanation has been stated at line 100-102.

5. Add precision around estimates.

Line 207: make it clear what the numbers are (presume standard deviation).

**Response**: Thank you for your suggestion. Now we have added some changes:

Line 205: with an average of 5.69 ± 4.80 drugs/child.

Line 207: Average use of non-antibiotic medications per AE was 1.72 (SD±0.99).

The regression: how does this add to your clinical understanding?
What is the distribution of the variable number of drug regimens? Is linear regression the best model? Are the assumptions met? Was patient included as a variable in the model? Is it modelling how many drug regimens at a given time (or age) or overall from 0-18 months in a child’s life? This is a little unclear.

**Response:** We thank the reviewer for noting this issue. After re-considering the best model to explore the factors that influenced the number of drug regimen in our study, we decided that instead of using a linear regression as in our previous manuscript, a logistic regression is better to provide the RR. Therefore, at the following lines we have added some changes:

Line 230-238: “A logistic regression was performed to explore the relationship between the reason of medication use, drug formulation, outpatient/inpatient setting, and the age of participant when using the medication with the number of non-antibiotic drug regimen.“

We also have revised the Table 4 and lines 348-355 (discussion section).

6. Table 7 include precision estimates.

How is 1518 people 100% when there were over 1600 patients on study? Did the others not have AES, or just not have it recorded? This is an important distinction to be made.

**Response**: Thank you for noting this. At table 7, we only included participants who had at least one AE during the study, therefore 1518 (of 1621) was 100%. Now we have added this information under the table.

“The table included only participants who had at least one AE during the 18 months of follow-up (N=1518/1621). The list is ordered by the proportion of adverse event (AE) episodes treated with non-antibiotic medications. SE: Standard Error of proportion.”

7. Line 300. Add a .

Response: Thank you for noting this, we have added . at line 300 (now at line 301).

8. Line 389 and 390. How big were these 2 studies? If they were small there may not be a significant difference in the rates.

Response:

Thank you for your suggestion, now we have revised the articles at line 390-393 by adding the total children of each study:

“Half of our participants used at least one systemic antihistamine during their first 18 month of life, which is higher compared to other studies showing 31% of **4511** children had used antihistamines by the age of 2 years in the Netherlands [1], and 17% of **1701** children during the first 5 years of life in Sweden [20].”

9. Figure 2 and 3- each patient could contribute multiple times to each result couldn’t they?

Response: Yes, each patient could contribute multiple times to each result. Now we have added this information under the figure legend. Thank you.
