# Supplementary material for: Non-antibiotic medication use in an Indonesian community cohort 0–18 months of age
Source: PLoS One. 2020 Nov 18;15(11):e0242410. doi: 10.1371/journal.pone.0242410 (PMC7673523; doi:10.1371/journal.pone.0242410)
Supplement: Attachment — Submitted filename: Response to reviewers_3rd revision (ver3.1_26Sept2020).docx [file pone.0242410.s007.docx]

October 1, 2020

Subject: Submission of revised paper *Non-antibiotic Medication Use in an Indonesian Community Cohort 0-18 Months of Age*

[Manuscript number: PONE-D-19-26757]

Dear Academic Editor and Reviewers

Thank you for spending your valuable time to read our previously revised manuscript and keep giving insightful suggestions to help us improve the quality of our manuscript.

Along with this letter are the Reviewers comments, followed by our point-by-point response in addition to making the changes in the revised manuscript. Responses to the Reviewers’ comments are typed in blue font, and the changes made in the manuscript are marked using track changes. Each author has given approval to the final form of this revision.

We believe that the revision on our manuscript will improve the quality of our paper. We look forward to hearing from you in due course.

Sincerely,

Jarir At Thobari, MD, DPharm, PhD

Department of Pharmacology and Therapy and Pediatric Research Office (PRO)

Faculty of Medicine, Public Health, and Nursing

Universitas Gadjah Mada

On behalf of all authors

**I. Academic Editor comment**

2. I can see some alterations to the paper, which assist with clarity of understanding. There are a few other areas for consideration before this could be published.

In the statistical analysis section line 185 of track changed doc it states that "descriptive data, the results were presented as mean, median, frequency and percentages". Then on line 207 it states, "an average of 5.69 +/- 4.80 drugs/child". Is the 4.80 the median? If not, please explain what this is, and/or change the statistical analysis methods.

**Response:** Thank you for your suggestion. The 4.80 is a standard deviation of the mean (5.69). We have revised the “average” on line 205 into “a mean (SD)”.

**II. Reviewer #1 comments**

1. Table 5: Row 5 and row 14- what does anti-infective agents in row 5 mean? is it antibiotics for GI infections. If it is antibiotics then it should be removed as table is showing non antibiotic medications. Similarly, I believe antiseptics and disinfectants in row 15 means topical agents

**Response**: We apologize for the ambiguity in table %. In Table 5 Row 5, we used the exact ATC WHO therapeutic class name of “antidiarrheals, intestinal anti-inflammary /anti-infective agents”. However, we have already removed the anti-infective drugs from the very beginning. Therefore, to avoid this ambiguity, now we have revised the therapeutic class name in Table 5 Row 5 as “antidiarrheals and intestinal antiinflammary agents” only.

In Table 5, Row 14, indeed, antiseptics and disinfectants are topical agents. Now we have added such information in Table 5 Row 14. This additional information is also for addressing the reviewer 3rd comment “ear disorders – antiseptics and disinfectants are topical preparations only?”

2. Table 5: Row 7 “drugs for obstructive airway disease” I believe are bronchodilators. Bronchodilators gives a better understanding for these drugs.

Table 5: Row 8 –are these corticosteroids for topical use only?

**Response:** Row 7: Yes, this therapeutic class of ATC WHO contains any bronchodilator types. We have revised it into “Bronchodilators” as your suggestion. Thank you.

In Row 8 – Yes, the corticosteroids for dermatological preparations are topical agents as well. Now we have revised it into “corticosteroids (topical agents)”. Thank you for noting this.

3. Figure 3: Ear disorders – antiseptics and disinfectants are topical preparations?

**Response**: We have added an additional information to address this suggestion on Table 5 Row 14.

4. Line 343-344: reformatting of sentence as " This also explains a usage/consumption of higher number of drugs by older infants...something like that

**Response**: Thank you. We have reformatted the sentence as your suggestion on line 342-343: “This also explains a consumption of higher number of drugs by older infants (Table 4).”

5. Line 345-346.Reformatting of sentence the inclusion of prescription and non-prescription drugs and the exclusion of antibiotics in our study.....

**Response:** Thank you. We have reformatted the sentence as your suggestion on line 344: “The inclusion of prescription and non-prescription drugs and the exclusion of antibiotics in our study also might affect the average number of drug regimen compared to those who calculated prescribed drugs only without excluding antibiotic, as non-prescribed drug tend to be used in milder conditions that need fewer number of drug per regimen.”

6. Line 352,353. reformatting of sentence. Respiratory disorders, gastrointestinal disorders and unspecified pyrexia manifest as multiple symptoms....

**Response**: Thank you. We have reformatted the sentence as your suggestion on line 351: “Respiratory disorders, gastrointestinal disorders, and unspecified pyrexia manifest as multiple symptoms, thus may encourage child caretakers to self-medicate the children with multiple over-the-counter (OTC)”

7. Line 364.sentence formatting. Similar to other studies, respirator system disorder was the primary indication for medication use in our study

**Response**: Thank you. We have reformatted the sentence as your suggestion on line 361-362: “Similar to other studies, respiratory system disorder was the primary indication for medication use in our study”.

8. Line 371-373.sentence formatting Analgesics/antipyretics....sentence is not very clear. This drug usage pattern was similar to other studies

**Response**: Thank you. We have reformatted the sentence as your suggestion on line 370: “This drug usage pattern was similar to other studies”

9. Line 378s-seems like you are comparing the same age group population.it does not seem different in age. Please clarify

**Response**: The age of our participants ranged from 0-18 months, compared to the age of 0-18 years in the other study. This might sound similar and confuse the reader, therefore we have reformatted the sentence in line 375 into: “The reasons for different results were difficult to determine, but possible explanations might be due to the younger age of participants in our study.”

10. Line 386-what CCMs

**Response**: Thank you for noting this. CCMs is an abbreviation of cough and cold medications. We have added this information in the first abbreviation in line 384.

11. Line 396..sentence formatting Higher incidence of respiratory tract infections

**Response**: Thank you. We have reformatted the sentence in line 393-394 as your suggestion into “This might partly reflect a higher incidence of respiratory tract infections in our population.”

12. Line 408-409.Its a better idea to mention percent of deaths from each drug

**Response**: Thank you for your suggestion. We have put the percentage of deaths from each drug in line 405-406.

13. Line 427.sentence formatting Although not being investigated in children.

**Response**: Thank you. We have reformatted the sentence in line 424 as your suggestion into: “Although not being investigated in children, vitamin B6 overdose in adult has been known to induce neurotoxic syndrome”.

14. Conclusion paragraph reformatting The majority of children were exposed.....A variety of medications were used.....This calls for further education....

**Response**: Thank you. We have reformatted the sentence in conclusion paragraph (line 442) as your suggestion into: “The majority of children **were** exposed to at least one medication during their early life. **A variety of medications were used,** including those where the evidence of efficacy and safety in pediatric population is lacking. **This calls for further education** to improve caregivers’ knowledge on appropriate self-medication in children, and to enhance the guideline compliance by health practitioners to ensure the therapy given to children will be of most benefit.“
